# Supplementary material for: Caspase-2 promotes AMPA receptor internalization and cognitive flexibility via mTORC2-AKT-GSK3β signaling
Source: Nat Commun. 2019 Aug 9;10:3622. doi: 10.1038/s41467-019-11575-1 (PMC6689033; doi:10.1038/s41467-019-11575-1)
Supplement: Supplementary file 1 — Supplementary Information [file 41467_2019_11575_MOESM1_ESM.pdf]

SUPPLEMENTARY FIGURES

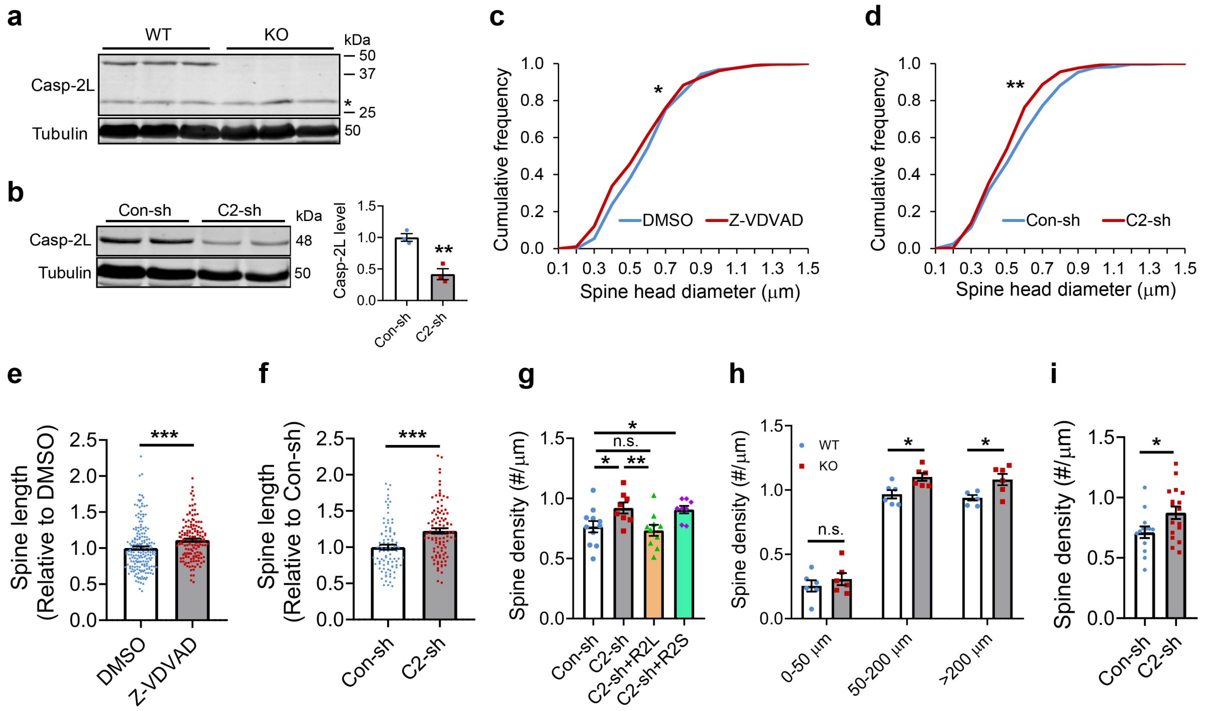

Supplementary Fig. 1. Caspase-2 deficiency increases spine density. a, Western blot showing specificity of caspase-2 antibodies. Rabbit antibodies against caspase-2 detected caspase-2 (48 kDa) and a non-specific protein (*) in mouse brain lysates. **b**, Knockdown efficiency of *Casp2* shRNA. Rat hippocampal neurons were infected with lentivirus expressing Con-sh (control shRNA) or C2-sh (*Casp2* shRNA) on DIV5 and harvested on DIV21. The efficiency of lentiviral infection was ~80%. n = 3 per condition. **c-f**, Effects of caspase-2 inhibition or knockdown on spine head size and length in cultured rat hippocampal neurons. Neurons were transfected with a construct expressing EGFP-actin on DIV14. On DIV21, they were treated with the caspase-2 inhibitor Z-VDVAD-FMK (10 μM) or DMSO for 7 days. Neurons were fixed for analysis of spine diameter (c) and length (e) on DIV28. Alternatively, neurons were transfected with constructs expressing Con-shRNA (Con-sh) or *Casp2* shRNA (C2-sh) on DIV14. To visualize spine morphology, neurons were co-transfected with a construct expressing EGFP-actin. Neurons were fixed for analysis of spine diameter (d) and length (f) on DIV28. DMSO, 369 spines from 13 neurons; Z-VDVAD, 308 spines from 11 neurons; Con-sh, 281 spines from 10 neurons; C2-sh, 292 spines from 10 neurons. Data were analyzed using nonparametric multiple comparison with Mann–Whitney test (c, d) or two-tailed Student's *t* test (e, f). **g**, Overexpression of shRNA-resistant caspase-2L, but not caspase-2S, normalized spine density in cultured rat hippocampal neurons expressing *Casp2* shRNA. Con-sh, control shRNA; C2-sh, *Casp2* shRNA; R2L, C2 shRNA-resistant mutated long-form *Casp2* mRNA; R2S, C2 shRNA-resistant mutated short-form *Casp2* mRNA. n = 11, 8, 10, and 8 neurons for Con-sh, C2-sh, C2-sh + R2L, and C2-Sh + R2S, respectively. One-way ANOVA with LSD post-hoc test. **h**, *Casp2* KO mice showed normal proximal but increased distal dendritic spine density in CA1 neurons. n = 6 mice per genotype. Data were analyzed using two-tailed Student's *t* test. **i**, Knockdown of caspase-2 increased spine density in dendritic segments used for time-lapse imaging. Con-sh, n = 14 neurons; C2-sh, n = 19 neurons. Data were analyzed using two-tailed Student's *t* test. Data are presented as mean ± SEM. n.s. = no significance, *p < 0.05, **p < 0.01, and ***p < 0.001. Source data are provided as a Source Data file.

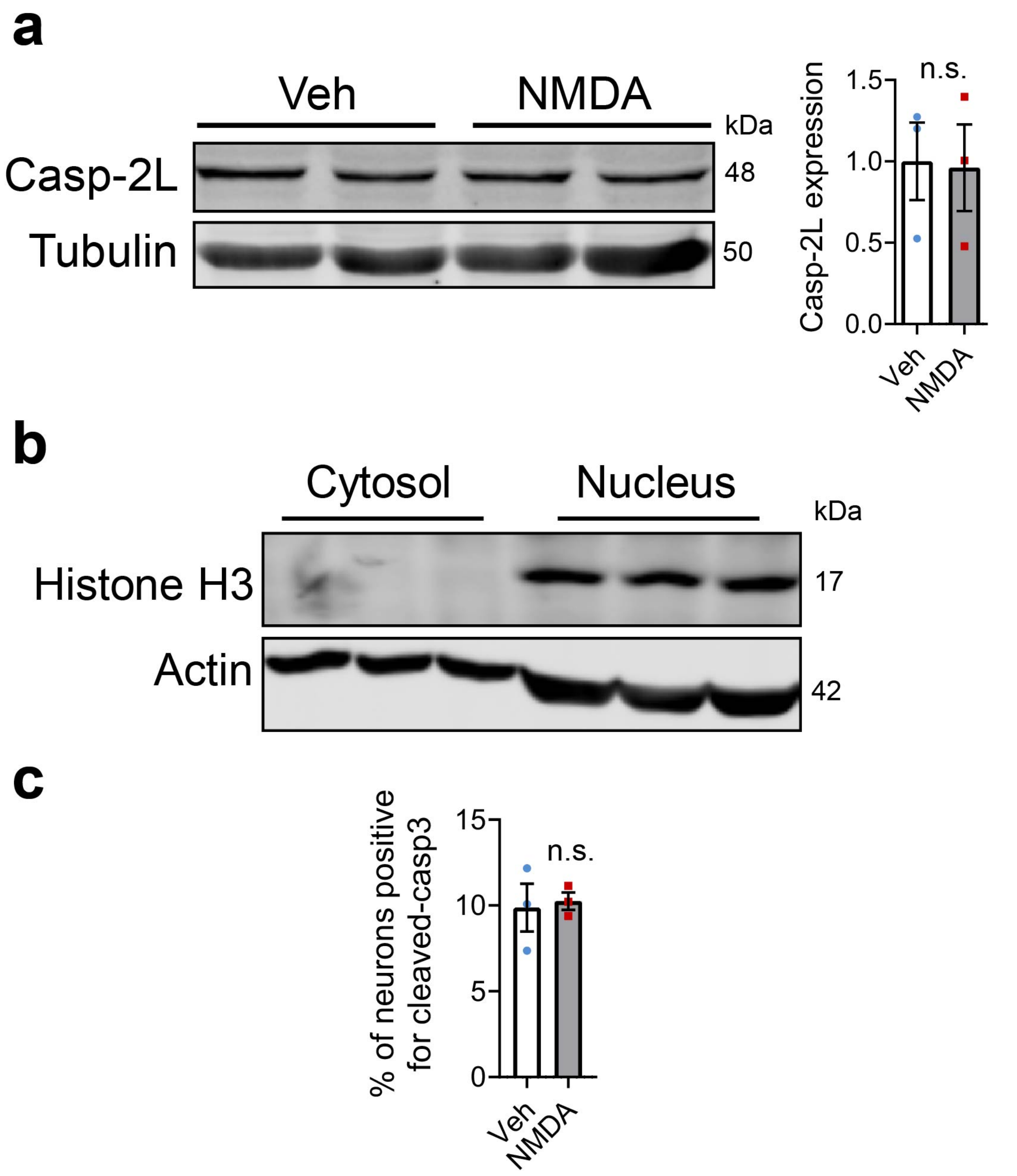

Supplementary Fig. 2. Activation of the NMDA receptor in neuronal culture. a, Western blot analysis revealing comparable amounts of caspase-2 in vehicle (Veh) and NMDA-treated cultures of mouse cortical neurons. **b**, Immunoblotting analysis of histone H3 in cytosolic and nuclear fractions prepared from cultured mouse cortical neurons. **c**, Brief NMDA treatment (40 µM for 5 minutes) doesn’t induce apoptosis in mouse hippocampal neuronal cultures. Veh and NMDA, n = 3. Data are presented as mean ± SEM. n.s., not significant by two-tailed Student’s *t* test. Source data are provided as a Source Data file.

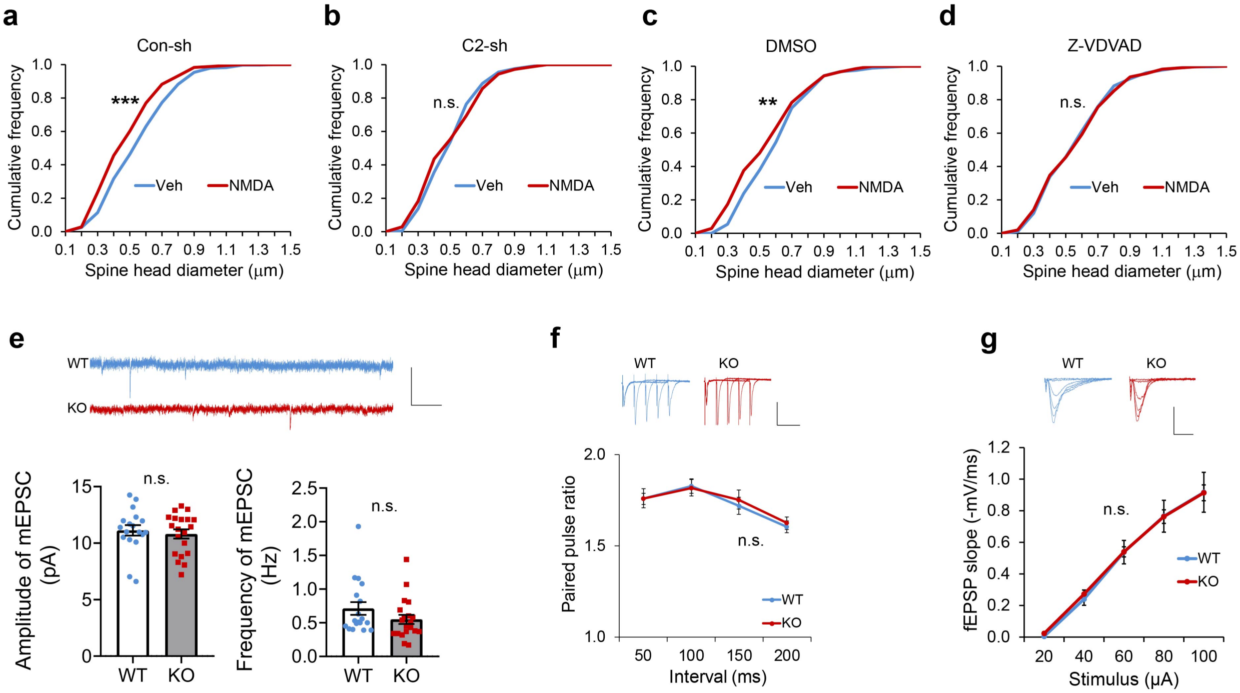

Supplementary Fig. 3. Caspase-2 deficiency blocks NMDA-induced spine shrinkage. a-d, Neurons were briefly treated with vehicle or 40 μM NMDA for 5 min on DIV28 and were fixed for analysis of dendritic spines 30 min after treatment. Con-sh and Veh, 281 spines from 10 neurons; C2-sh and Veh, 292 spines from 10 neurons; Con-sh and NMDA, 178 spines from 6 neurons; C2-sh and NMDA, 356 spines from 12 neurons; DMSO and Veh, 369 spines from 13 neurons; Z-VDVAD and Veh, 308 spines from 11 neurons; DMSO and NMDA, 208 spines from 7 neurons; Z-VDVAD and NMDA, 299 spines from 10 neurons. Nonparametric multiple comparison with Mann–Whitney test: n.s.= no significance, $**p < 0.01$ and $***p < 0.001$. **e**, Amplitude and frequency of mEPSCs in CA1 pyramidal neurons are comparable between WT and *Casp2* KO mice. WT, 18 cells from 5 mice; KO, 20 cells from 6 mice. Scale bars, 20 pA (vertical) and 0.5 s (horizontal). Not significant (n.s.) by two-tailed Student's *t* test. **f**, Normal paired pulse ratio at Schaffer collateral-CA1 synapses of *Casp2* KO mice. WT, 12 slices from 6 mice; KO, 18 slices from 9 mice. Scale bars, 1 mV (vertical) and 100 ms (horizontal). Not significant (n.s.) by one-way ANOVA with Bonferroni post-hoc test. **g**, Normal input–output curve at Schaffer collateral-CA1 synapses of *Casp2* KO mice. WT, 30 slices from 12 mice; KO, 36 slices from 16 mice. Scale bars, 1 mV (vertical) and 10 ms (horizontal). Not significant (n.s.) by one-way ANOVA with Bonferroni post-hoc test. Data in panels e-g are presented as mean ± SEM. Source data are provided as a Source Data file.

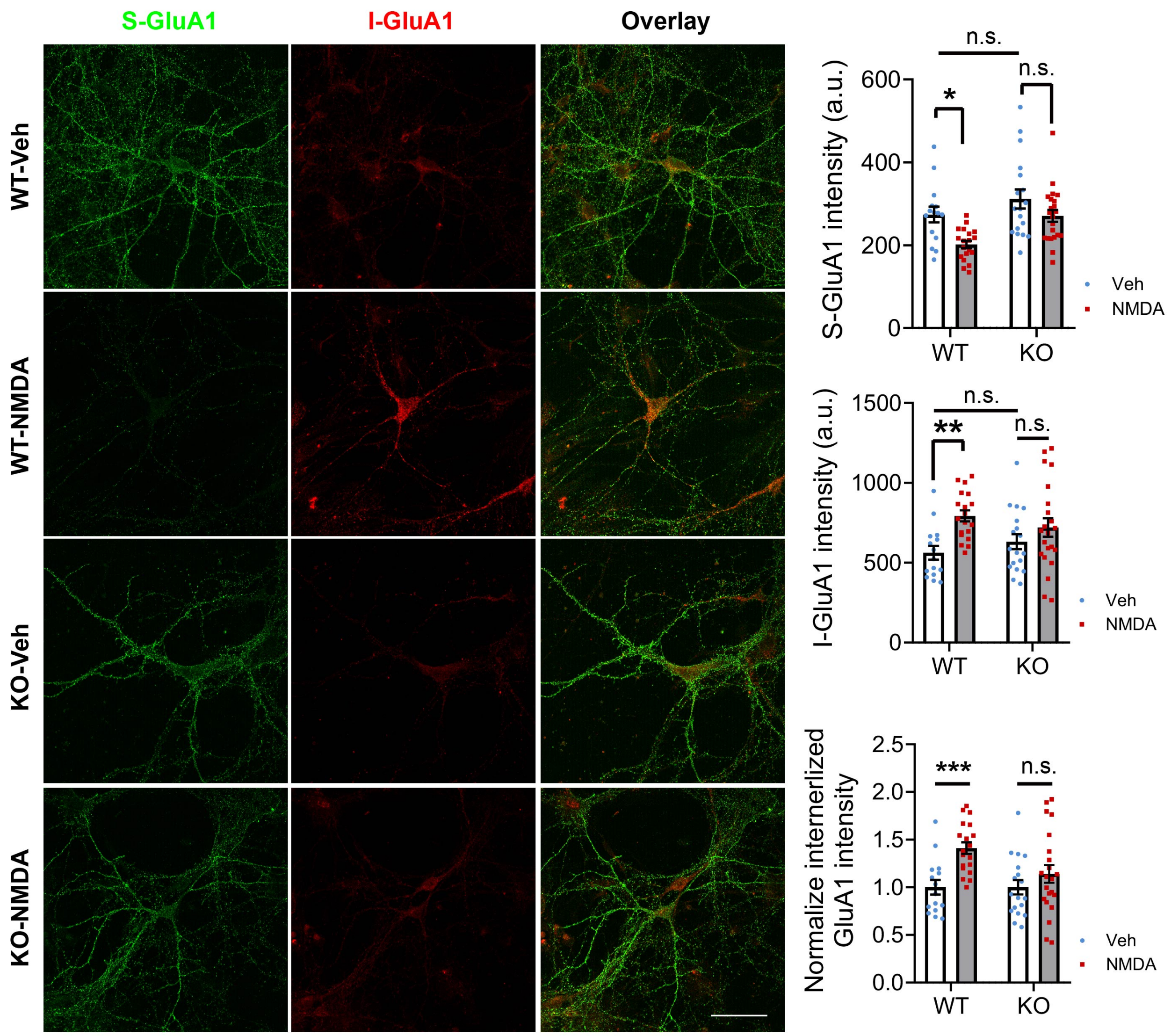

Supplementary Fig. 4. Internalization of surface GluA1 upon induction of chemical LTD. Representative images of surface (S-GluA1, green) and internalized (I-GluA1, red) GluA1 in cultured mouse hippocampal neurons. In WT neurons, brief NMDA treatment reduced surface GluA1, while increasing internalized GluA1. The treatment did not change levels of S-GluA1 and I-GluA1 in *Casp2* KO neurons. Histograms show levels of S-GluA1, I-GluA1 and normalized internalized GluA1 (relative to vehicle-treated WT neurons) for each genotype. Data are presented as mean ± SEM. The scale bar represents 50 μm. One-way ANOVA with Bonferroni post-hoc test: n.s. = no significance, *p < 0.05, **p < 0.01, and ***p < 0.001, n = 15-22 neurons each group. Source data are provided as a Source Data file.

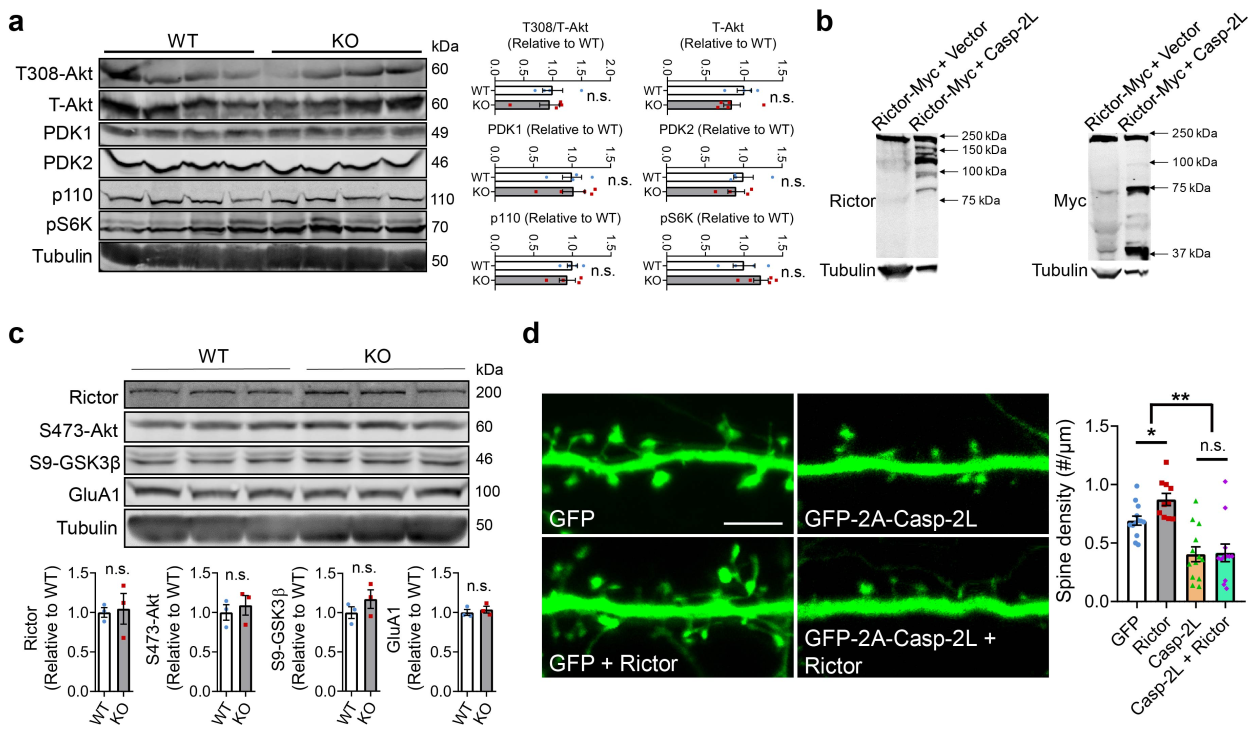

Supplementary Fig. 5. Regulation of Akt activity by caspase-2. a, Levels of Thr308-Akt, total Akt, PDK1, PDK2, p110 and pS6K in the hippocampus of WT and *Casp2* KO mice. n = 4-5 per genotype. n.s., not significant by two-tailed Student's *t* test. **b**, Immunoblotting analysis of Rictor cleavage products in HEK293 cells using Rictor antibodies (recognizing N-terminal cleavage products) and Myc antibodies (recognizing C-terminal cleavage products). Protein extracts were prepared 48 hours after transfection. **c**, Immunoblotting analysis and quantification of Rictor, Ser473-Akt, Ser9-GSK3β and GluA1 in cultured WT and *Casp2* KO neurons. n = 3 per genotype. n.s., not significant by two-tailed Student's *t* test. **d**, Rat hippocampal neurons were transfected with constructs expressing either GFP, GFP-2A-Caspase-2L, GFP + Rictor, or GFP-2A-Casp-2L + Rictor on DIV7. Projected images from stacks of optical sections were used for analysis of spine density in DIV19 neurons. One-way ANOVA with LSD post-hoc test: n.s. = no significance, $*p < 0.05$, $**p < 0.01$, n = 10-13 neurons for each group. Data are presented as mean ± SEM. Source data are provided as a Source Data file.

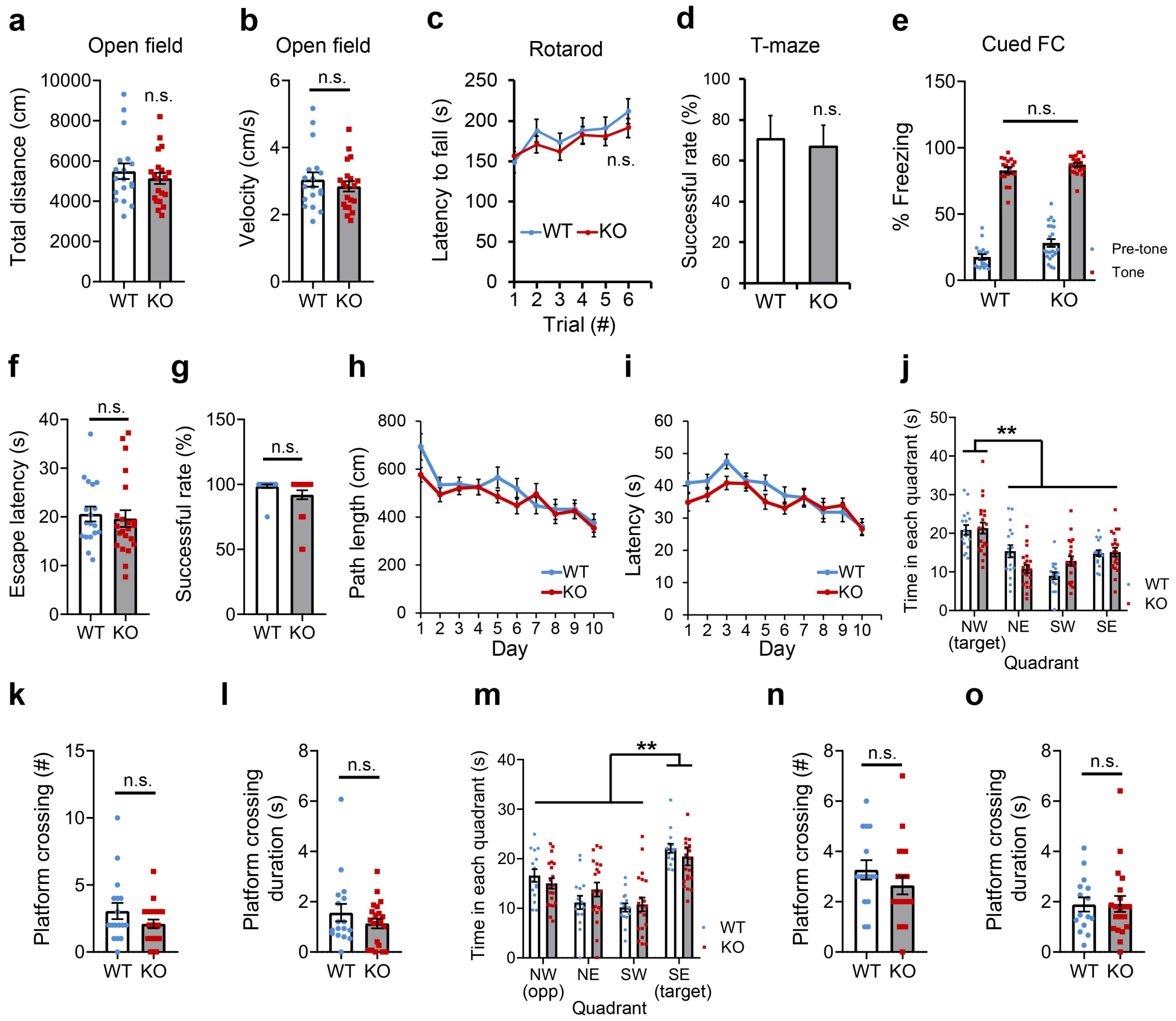

Supplementary Fig. 6. Locomotion- and memory-related behaviors of *Casp2* KO mice. a, Total distance WT and KO mice traveled in open field tests. **b**, Travel velocity of WT and KO mice in open field tests. **c**, Performance of WT and KO mice in accelerating rotarod rod tests. **d**, Short-term memory revealed by T maze alternation tests. **e.** Cued fear conditioning (FC) memory was indistinguishable between WT and KO mice. **f**, **g**, WT and KO mice had comparable performance in the visual platform version of MWM test. **h**, Swimming path of WT and KO mice in the hidden platform version of MWM test. All training days (except for day 1), $p > 0.05$ by two-way ANOVA with Bonferroni post-hoc test. **i**, Escape latency of WT and KO mice in the hidden platform version of MWM test. All training days, $p > 0.05$ by two-way ANOVA with Bonferroni post-hoc test. **j-l**, Performance at the first probe trial on day 11. WT and KO mice spent comparable amount of time in the target quadrant. They crossed the location of the hidden platform with similar numbers and duration. **m-o**, Performance at the second probe trial on day 17 after reversal training. WT and KO mice spent comparable amount of time in the target quadrant. They crossed the location of the hidden platform with similar numbers and duration. Data are presented as mean ± SEM. Data were analyzed using two-tailed Student's *t* test unless a statistical method is specifically stated. n = 15-22 mice per genotype; n.s. = $p > 0.05$, $^{*}p < 0.05$ and $^{**}p < 0.01$. Source data are provided as a Source Data file.
