# Supplementary material for: Caspase-2 promotes AMPA receptor internalization and cognitive flexibility via mTORC2-AKT-GSK3β signaling
Source: Nat Commun. 2019 Aug 9;10:3622. doi: 10.1038/s41467-019-11575-1 (PMC6689033; doi:10.1038/s41467-019-11575-1)
Supplement: Supplementary file 3 — Reporting Summary [file 41467_2019_11575_MOESM3_ESM.pdf]

natureresearch

Corresponding author(s): Baoji Xu

Reporting Summary

Nature Research wishes to improve the reproducibility of the work that we publish. This form provides structure for consistency and transparency in reporting. For further information on Nature Research policies, see Authors & Referees and the Editorial Policy Checklist.

Statistical parameters

When statistical analyses are reported, confirm that the following items are present in the relevant location (e.g. figure legend, table legend, main text, or Methods section).

n/a	Confirmed	
☐	☒	The exact sample size (*n*) for each experimental group/condition, given as a discrete number and unit of measurement
☐	☒	An indication of whether measurements were taken from distinct samples or whether the same sample was measured repeatedly
☐	☒	The statistical test(s) used AND whether they are one- or two-sided<br>*Only common tests should be described solely by name; describe more complex techniques in the Methods section.*
☐	☒	A description of all covariates tested
☐	☒	A description of any assumptions or corrections, such as tests of normality and adjustment for multiple comparisons
☐	☒	A full description of the statistics including central tendency (e.g. means) or other basic estimates (e.g. regression coefficient) AND variation (e.g. standard deviation) or associated estimates of uncertainty (e.g. confidence intervals)
☐	☒	For null hypothesis testing, the test statistic (e.g. *F*, *t*, *r*) with confidence intervals, effect sizes, degrees of freedom and *P* value noted<br>*Give P values as exact values whenever suitable.*
☒	☐	For Bayesian analysis, information on the choice of priors and Markov chain Monte Carlo settings
☒	☐	For hierarchical and complex designs, identification of the appropriate level for tests and full reporting of outcomes
☒	☐	Estimates of effect sizes (e.g. Cohen's *d*, Pearson's *r*), indicating how they were calculated
☐	☒	Clearly defined error bars<br>*State explicitly what error bars represent (e.g. SD, SE, CI)*

Our web collection on statistics for biologists may be useful.

Software and code

Policy information about availability of computer code

Data collection	Image Studio Lite Ver 4.0 (Western blot); Neurolucida software (Spine density); NIS-Elements Confocal (Immunoflourescence); pCLAMP 10 (Electrophysiology); EthoVision XT (Behavior)
Data analysis	Image Studio Lite Ver 4.0 (Western blot); Neurolucida software (Spine density); NIS-Elements Advanced Research (Immunoflourescence); Mini Analysis software (mEPSC); EthoVision XT (Behavior); SPSS (Statistics)

For manuscripts utilizing custom algorithms or software that are central to the research but not yet described in published literature, software must be made available to editors/reviewers upon request. We strongly encourage code deposition in a community repository (e.g. GitHub). See the Nature Research guidelines for submitting code & software for further information.

Data

Policy information about availability of data

All manuscripts must include a data availability statement. This statement should provide the following information, where applicable:

- Accession codes, unique identifiers, or web links for publicly available datasets
- A list of figures that have associated raw data
- A description of any restrictions on data availability

The data that support the findings of this study are available from the corresponding author upon reasonable request. Source data for all figures are provided with the paper.

Field-specific reporting

Please select the best fit for your research. If you are not sure, read the appropriate sections before making your selection.

☒ Life sciences ☐ Behavioural & social sciences ☐ Ecological, evolutionary & environmental sciences

For a reference copy of the document with all sections, see nature.com/authors/policies/ReportingSummary-flat.pdf

Life sciences study design

All studies must disclose on these points even when the disclosure is negative.

Sample size	No sample-size calculation was performed. The sample size were selected based on published studies (An et al. cell, 2008; Orefice et al. JNS, 2013; Gkogkas et al. Nature 2013; Li et al. cell, 2010)
Data exclusions	Outliers for behavioral testing were identified as being greater than 2.5 standard deviations from the mean and excluded from statistical analysis. For Morris water maze behavioral test, mice that continuously floated and did not search actively for the escape platform were excluded.
Replication	All attempts at replication were successful.
Randomization	For molecular studies, culture neurons were randomly assigned to vehicle and NMDA treatments.
Blinding	For In vitro and in vivo spine density measurements, the experimenter was blind to genotype or treatment. All behavioral tests were performed with automated tracking systems.

Reporting for specific materials, systems and methods

Materials & experimental systems

n/a	Involved in the study	
☒	☐	Unique biological materials
☐	☒	Antibodies
☐	☒	Eukaryotic cell lines
☒	☐	Palaeontology
☐	☒	Animals and other organisms
☒	☐	Human research participants

Methods

n/a	Involved in the study	
☒	☐	ChIP-seq
☒	☐	Flow cytometry
☒	☐	MRI-based neuroimaging

Antibodies

Antibodies used	rabbit anti-cleaved caspase-3 (CST, 9661), mouse anti-HA (Covance MMA-101P), mouse anti-a-tubulin (Sigma-Aldrich T6074), mouse anti-Psd95 (Thermo Fisher Scientific MA1-045), rabbit anti-caspase-2 (Abcam ab179519), mouse anti-b-actin (Sigma-Aldrich A5441), mouse anti-synaptophysin (Abcam ab8049), rabbit anti-GluA1 (Millipore AB1504), mouse anti-GluA2 (Millipore MABN71), mouse anti-GluA3 (Millipore MAB5416), mouse anti-GluN1 (Millipore MAB363), mouse anti-PDK1 (Santa Cruz sc-293160), mouse anti-PDK2 (Santa Cruz sc-517284), mouse anti-p110 (Santa Cruz sc-8010), mouse anti-Rictor (Santa Cruz sc271081), rabbit anti-S473-AKT (CST 2920), rabbit anti-T308-AKT (CST 9275), mouse anti-pan AKT (CST 9271), rabbit anti-GSK3b (CST 9315), rabbit anti-Ser9-GSK3b (CST 9331), rabbit anti-mTOR (CST 2972), rabbit anti-pS6K (CST 9205), rabbit anti-GFP (Clontech 632592), rabbit anti-Histone H3 (CST 4499), rabbit anti-MAP2 (Santa Cruz Biotechnology #sc-20172)

Validation	All antibodies used are commercially available and validated.

Eukaryotic cell lines

Policy information about cell lines

Cell line source(s)	HEK293 cells were from ATCC
Authentication	The cell line used was not authenticated
Mycoplasma contamination	Not tested
Commonly misidentified lines (See ICLAC register)	None

Animals and other organisms

Policy information about studies involving animals; ARRIVE guidelines recommended for reporting animal research

Laboratory animals	Animals used in this study includes Sprague Dawley rats and C57BL/6 mice. Ages of animal were specified on methods for different assays. Animal care and experimental procedures were approved by Scripps Florida Institutional Animal Care and Use Committee (protocol # 16-003), according to US National Institutes of Health Guidelines (Online Methods/mouse husbandry).
Wild animals	did not involve wild animals.
Field-collected samples	did not involve samples collected from the field.
